# Systemic inflammatory markers and EEG features of children with FIRES receiving anakinra

**DOI:** 10.1002/acn3.51714

**Published:** 2023-01-16

**Authors:** Yi‐Chen Lai, Gabriella Abou‐El‐Kheir, Thao Nguyen, Margo Hanerhoff, James J. Riviello, Eyal Muscal

**Affiliations:** ^1^ Division of Pediatric Critical Care Medicine Baylor College of Medicine Houston Texas USA; ^2^ Texas Children's Hospital Houston Texas USA; ^3^ Division of Neurology and Developmental Neuroscience, Department of Pediatrics Baylor College of Medicine Houston Texas USA; ^4^ Division of Pediatric Rheumatology, Department of Pediatrics Baylor College of Medicine Houston Texas USA; ^5^ Present address: Division of Pediatric Critical Care Medicine, Department of Pediatrics UT Health System McGovern Medical School Houston Texas USA

## Abstract

In a retrospective case series of 10 children with cryptogenic FIRES, we sought to describe the early clinical course and potential biomarkers following anakinra initiation. Six children achieved anesthetic withdrawal within 3 weeks of therapy and one in week four. Of the available cEEG (six children), CRP (10 children), and serum cytokine (six children) studies, there were temporal changes in highly epileptiform bursts (observed in three children), CRP, IL‐6, and IL‐10 levels that might parallel clinical progression. These observations may represent candidate biomarkers for monitoring clinical progression and therapeutic interventions including anakinra, which merits further investigation in future studies.

## Introduction

Febrile‐infection‐related epilepsy syndrome (FIRES) is a subcategory of new‐onset refractory status epilepticus (NORSE) that is preceded by a febrile illness.^1^ Withdrawal of the anesthetic infusions for seizure control often leads to the re‐emergence of high seizure burden, which contributes to a protracted intensive care unit course with prolonged mechanical ventilation days and significant in‐hospital mortality.^2^, ^3^, ^4^

Although the etiologies underpinning FIRES remain elusive, increasing evidence support neuroinflammation as a potential pathological mechanism.^5^, ^6^, ^7^, ^8^ Specifically, aberrant innate immunity activation and/or functional deficiency of the endogenous interleukin‐1 receptor antagonist (IL‐1ra) have been described in children with FIRES.^5^, ^6^ Accordingly, exogenous IL‐1ra (anakinra) administration is increasingly utilized and proposed as a potential therapeutic option for FIRES.^5^, ^6^, ^9^, ^10^, ^11^, ^12^ In an international retrospective cohort of children with FIRES, anakinra therapy was found to be safe and potentially effective.^11^

Despite these promising results, there is limited information regarding the early clinical progression and characteristics following anakinra initiation. Therefore, we sought to describe the early clinical course of 10 consecutive children with FIRES following anakinra treatment.

## Subjects and Methods

The Baylor College of Medicine Institutional Review Board approved this retrospective care series with a waiver of consent. Children were included in the study if they had cryptogenic FIRES^1^ and received anakinra. We collected patient demographics, clinical, and laboratory information including cerebrospinal fluid (CSF) neopterin and cytokine profiles, serum c‐reactive protein (CRP), and cytokine profiles. Furthermore, we collected the anesthetic infusion and anti‐seizure medication (ASM) regimen 24 h prior to, 1 week, 2 weeks, and 3 weeks following anakinra initiation. Complete cEEG recordings were available for five children, and one child had an incomplete set of cEEG studies. A neurophysiologist (JR) who was blinded to the anakinra treatment epoch retrospectively reviewed cEEG recordings according to the American Clinical Neurophysiology Society terminology definitions.^13^ Seizure frequency was expressed as the number of seizures per 24 h. We semi‐quantified the frequency of generalized and lateralized periodic discharges (GPD/LPDs) and epileptiform discharges.^13^ Highly epileptiform bursts (HEBs) were defined as present if two or more epileptiform discharges occurred within >50% of bursts and at an average of 1 Hz or faster within a single burst. We generated only descriptive statistics due to the small sample size.

## Results

From 2017 to 2020, 10 consecutive children with FIRES were treated with anakinra. Six children in the current study were included in a prior international case series.^11^ Demographic and clinical information are summarized in Table 1. All children required anesthetic infusions for seizure control, most commonly midazolam, and pentobarbital. All children received steroid and intravenous immunoglobulin as per accepted standards of care.^12^ Six were on the ketogenic diet. The median time from seizure onset to anakinra initiation was 20.5 [10–28] days. The median maximum dosage was 7.5 [4.2–7.8] mg/kg/d.

**Table 1:** Patient demographics and clinical information.

Subject		1	2	3	4	5	6	7	8	9	10
Age (years)		8.9	12.4	5.6	6.9	6.6	4.7	14.9	15.8	17	7.3
Gender		M	M	F	M	M	M	F	M	F	M
Ethnicity		AA	Middle Eastern	H	H	H	W	A	H	H	A
Anakinra starts from seizure onset (days)		17	41	28	28	5	30	10	23	18	8
Initial anakinra (mg/kg/d)		3.2	3.8	4.7	8.7	3.9	3.8	5	2.8	4.2	3.2
Max anakinra (mg/kg/d)		3.2	7.5	10	8.7	7.8	7.5	7.5	4.2	4.2	6.4
Anakinra duration		91	162	199	256	22	964	662	562	291	274
*Summary of anesthetic infusions, immune modulating therapies, and ketogenic diet during hospitalization*											
KTM (days)		0	98	5	0	21	5	0	0	0	0
MDZ (days)		15	216	147	16	71	1	41	26	29	9
Pentobarbital (days)		0	26	109	10	24	37	17	25	18	11
Immune modulation		steroid, IVIG	steroid, IVIG	steroid, IVIG, PLEX, rituximab	steroid, IVIG, PLEX	steroid, IVIG	steroid, IVIG, PLEX	steroid, IVIG	steroid, IVIG, PLEX	steroid, IVIG, PLEX	steroid, IVIG, PLEX
Ketogenic diet		no	yes	yes	no	yes	yes	yes	no	no	no
*Number of scheduled anti‐seizure medications*											
24 h pre anakinra		4	2	2	4	4	3	2	3	4	3
1‐week post anakinra		4	2	3	5	4	3	3	4	4	4
2 weeks post anakinra		4	2	4	3	4	3	3	5	4	4
3 weeks post anakinra		3	4	6	4	4	3	3	4	5	4
*Concurrent immune therapies*											
24 h pre anakinra		steroids	none	none	none	steroids	KD	steroids	steroids	steroids, IVIG	steroids
1‐week post anakinra		steroids	none	none	steroids	KD	KD	steroids, KD	steroids	steroids	PLEX
2 weeks post anakinra		steroids	none	none	steroids	KD	KD	steroids, KD	steroids	steroids	none
3 weeks post anakinra		steroids	none	steroids	steroids	KD	KD	KD	steroids	steroids	none
*Number of electrographic seizures per 24 h*											
24 h pre anakinra		–	13	–	–	0	0	–	9	0	9
1‐week post anakinra		–	0	–	–	5	0	–	42	0	0
2 weeks post anakinra		–	1	–	–	0	0	–	–	0	0
3 weeks post anakinra		–	2	–	–	0	0	–	–	0	0
*Generalized/Lateralized periodic discharges*											
24 h pre anakinra		–	yes	–	–	no	no	–	yes	yes	yes
1‐week post anakinra		–	yes	–	–	yes	yes	–	yes	yes	yes
2 weeks post anakinra		–	yes	–	–	yes	no	–	–	yes	no
3 weeks post anakinra		–	yes	–	–	no	no	–	–	no	no
*Frequency of epileptiform discharges*											
24 h pre anakinra		–	50–90%	–	–	< 1%	10–50%	–	50–90%	50–90%	< 1%
1‐week post anakinra		–	10–50%	–	–	50–90%	10–50%	–	10–50%	10–50%	50–90%
2 weeks post anakinra		–	10–50%	–	–	50–90%	1–10%	–	–	none	1–10%
3 weeks post anakinra		–	10–50%	–	–	< 1%	1–10%	–	–	<1%	none
*Highly epileptiform bursts*											
24 h pre anakinra		–	yes	–	–	no	yes	–	no	no	no
1‐week post anakinra		–	yes	–	–	yes	yes	–	no	no	no
2 weeks post anakinra		–	yes	–	–	yes	no	–	–	no	no
3 weeks post anakinra		–	yes	–	–	yes	no	–	–	no	no
*Serum inflammatory markers* ^a^											
⇑ CRP	pre anakinra	1 (2)	–	–	11 (12)	1 (1)	2 (2)	1 (2)	11 (15)	1 (1)	0 (2)
	post anakinra	–	16 (16)	5 (11)	1 (11)	1 (1)	7 (7)	7 (9)	16 (17)	3 (7)	0 (8)
⇑ sIL‐2	pre anakinra	–	–	–	–	–	–	–	0 (3)	–	0 (1)
	post anakinra	–	2 (7)	0 (2)	–	1 (2)	–	–		–	–
⇑ IL‐6	pre anakinra	1 (1)	–	1 (1)	–	–	–	–	2 (4)	–	0 (1)
	post anakinra	–	0 (7)	0 (3)	–	0 (2)	–	–	0 (1)	–	1 (1)
⇑ IL‐10	pre anakinra	1 (1)	–	1 (1)	–	–	–	–	0 (4)	–	0 (1)
	post anakinra	–	7 (7)	0 (3)	–	2 (2)	–	–	1 (1)	–	0 (1)

Subjects 2, 3, and 5 required anesthetic infusions at 3 weeks following anakinra initiation.

–, data not available; CRP, c reactive protein; IL‐6, interleukin 6; IL‐10, interleukin 10; IVIG, intravenous immunoglobulins; KTM, ketamine; MDZ, midazolam; PLEX, plasmapheresis; sIL‐2, soluble interleukin 2 receptor.

^a^ The data are presented as number of abnormal values (number of studies).

**Figure 1:**
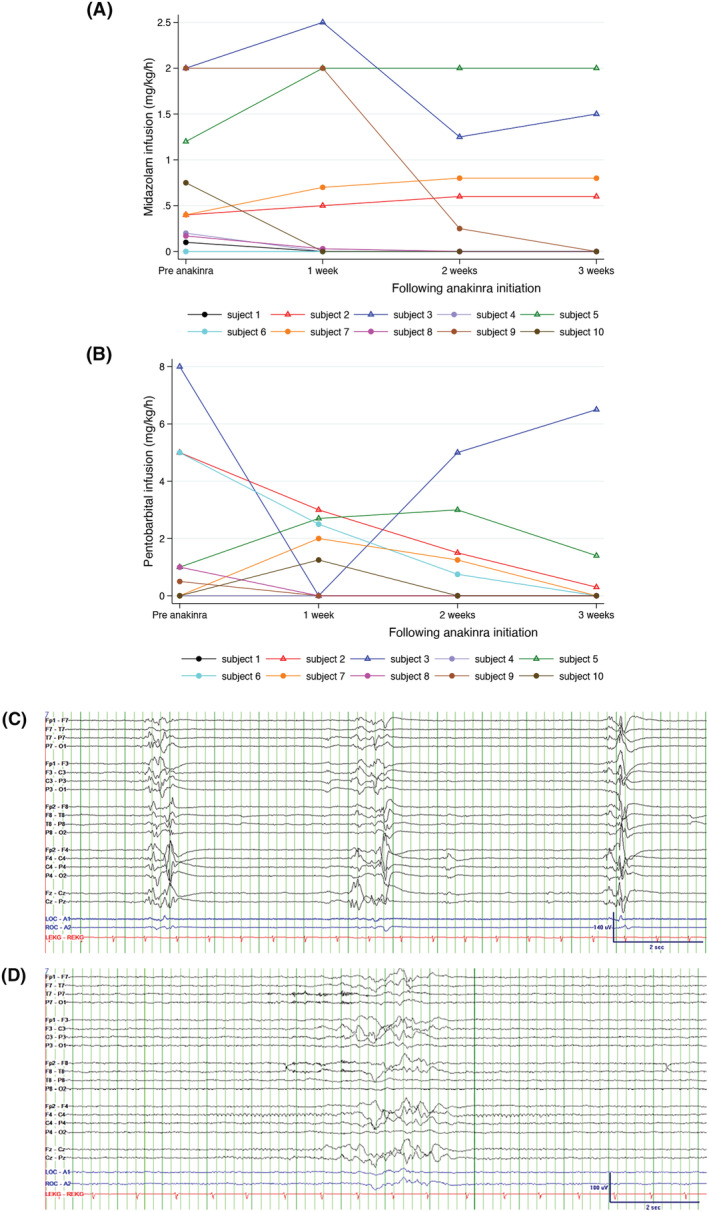
Changes in the anesthetic infusions following anakinra initiation. (A) Midazolam infusion rate of each subject at 24 h prior to, and 1 week, 2 weeks, and 3 weeks following anakinra initiation. (B) Pentobarbital infusion rate of each subject at the same time points. Closed circles: children with successful anesthetic withdrawal. Subject 7 (orange) was the child who had successful anesthetic withdrawal at week 4. Open triangle: children with ongoing anesthetic need at 3 weeks following anakinra. (C) EEG tracings of subject 6 before anakinra demonstrating discontinuous background with highly epileptiform bursts (HEBs). (D) EEG tracings of subject 6 following anakinra demonstrating discontinuous background with a burst of activities that are not HEBs.

Six children achieved complete anesthetic withdrawal within 3 weeks of anakinra initiation, and one child in week four (Fig. 1). Three children continued to require anesthetic infusions following 3 weeks of treatment. At week 3 of anakinra, six children were on more scheduled ASMs as compared with the pre‐anakinra period. We located cEEG studies for six children; two of whom remained on anesthetic infusions at week 3 of anakinra. Overall, seizures were identified in three children for 24 h prior to anakinra initiation. Two of the three had decreased seizures following anakinra initiation (Table 1). Of the three children who did not have seizures at the start of anakinra, two remained without seizures following anakinra administration (Table 1). There was an overall decreased prevalence of GPD/LPDs and epileptiform discharges following anakinra initiation (Table 1). Changes in seizures, GPDs/LPDs, and epileptiform discharges did not correlate with changes in anesthetic infusions. Nine cEEG studies from three children exhibited HEBs. HEBs persisted in two children and resolved in one (Table 1, Fig. 1). Children with persistent HEBs remained on anesthetic infusions at week 3 of anakinra.

**Figure 2:**
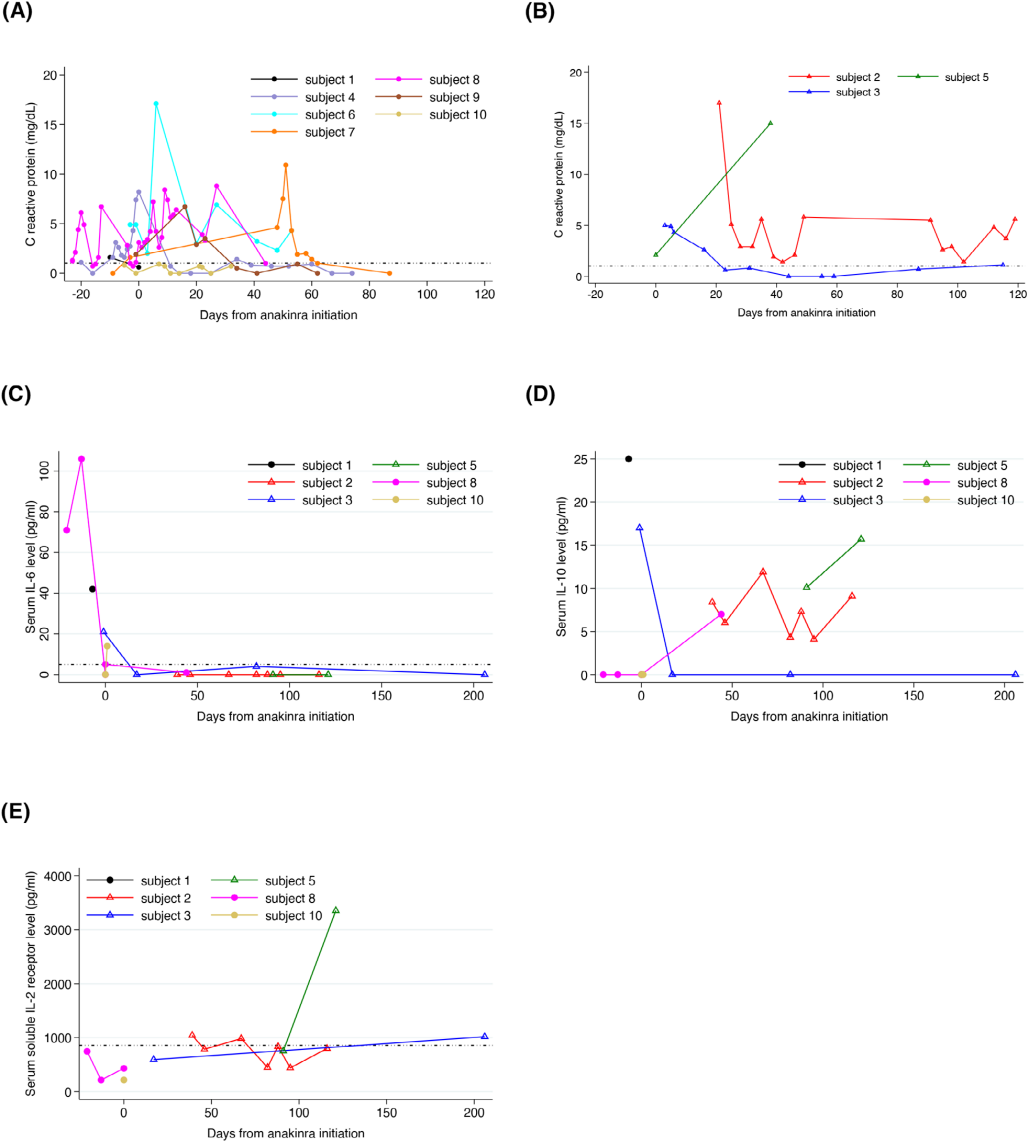
Serial systemic inflammatory markers in children with FIRES. (A and B) C‐reactive protein (CRP) levels of ten subjects, (C) interleukin 6 (IL‐6), (D) interleukin 10 (IL‐10), and (E) soluble interleukin 2 receptor (sIL‐2) levels of six subjects are plotted in relation to anakinra initiation. Closed circles: children with successful anesthetic withdrawal. Open triangles: children with ongoing anesthetic need at 3 weeks following anakinra. The dashed lines represent the upper limits of normal values for CRP and IL‐6 respectively. IL‐10 values that are below the upper limits of normal are assigned a value of “0” for ease of visualization.

There were three CSF cytokine profiles and four CSF neopterin studies following anakinra treatment. Two children had increased interleukin 2 (IL‐2) and 8 (IL‐8) levels in the CSF, and none had elevated neopterin levels. Serial serum measurements revealed a high prevalence of abnormal CRP levels (9 of 10 children) in the absence of an intercurrent infection, defined as negative bacterial and viral studies 7 days before and after a CRP, that remained elevated for a prolonged period (Fig. 2). Amongst children with successful anesthetic withdrawal, one had persistently elevated CRP (Fig. 2A). Two of three children with continued anesthetic need had persistently elevated CRP (Fig. 2B). Serum cytokine profiles that were obtained at variable time points in relation to anakinra initiation were available in six children (Table 1, Fig. 2). Elevated interleukin 6 (IL‐6) levels occurred in four children prior to anakinra (Fig. 2C). Elevated interleukin 10 (IL‐10) occurred in two children prior to, and three children after, anakinra treatment (Fig. 2D). Elevated soluble interleukin 2 receptor (sIL‐2) levels occurred in three children after anakinra initiation (Fig. 2E).

## Discussion

In this study, we described the initial course and clinical progression following anakinra initiation in 10 children with cryptogenic FIRES. Anakinra was temporally associated with the successful withdrawal of the anesthetic infusions in six children within 3 weeks and one child on week 4 of therapy initiation. We observed potential temporal changes in several parameters such as HEBs on cEEG, serum CRP and cytokine profiles. These observations raise an intriguing possibility that they may represent candidate biomarkers for the clinical progression, which may be used potentially for therapeutic monitoring and therefore merits further exploration in future studies.

We found a decrease in anesthetic infusions in most children occurring as early as the first week of anakinra treatment. This is consistent with the prevailing literature demonstrating decreased seizure burden with anakinra during the same period.^6^, ^11^ While the complete withdrawal of the anesthetic infusions occurred in some children within the first week of treatment, other children achieved anesthetic withdrawal in the second or third week of therapy. Therefore, 3 weeks of therapeutic anakinra trial may be needed to determine the full treatment response. Interestingly, children who had successful anesthetic withdrawal required lower anesthetic dosage at the start of anakinra, suggesting that there might be a differential treatment response based on the illness severity. Presently, the beneficial effects of anakinra are assessed primarily based on seizure burden or ability to withdraw anesthetic infusions.^6^, ^11^ However, a direct comparison of seizure frequency prior to and following anakinra initiation may be challenging in patients with burst‐suppression or with ictal‐interictal EEG patterns without definitive clinical or electrographic seizures as we observed in these children. Additionally, complete anesthetic withdrawal may simply reflect the replacement by scheduled ASMs. Therefore, other cEEG features such as HEBs may be a useful adjunct to monitor the clinical progression including the effects of anakinra.

Abnormal CRP values, a nonspecific systemic inflammatory marker,^14^ were common in this cohort, suggesting that systemic inflammation and an acute phase response may also be active in cryptogenic FIRES. This is consistent with the contemporary literature demonstrating active systemic inflammation in adults with status epilepticus, children with febrile status epilepticus, and children with FIRES.^6^, ^15^, ^16^ Interestingly, abnormal CRP in adults with status epilepticus has been shown to be independently associated with seizure refractoriness.^16^ Whether the changes in CRP values in this cohort similarly reflect seizure refractoriness and therefore may serve as a potential biomarker to monitor the clinical progression is currently unknown.

Elevated levels of pro‐inflammatory cytokines have been described in the CSF of patients with FIRES,^5^, ^6^, ^7^, ^8^ supporting immune activation of the central nervous system as a candidate pathological mechanism. In contrast, the serum cytokine profile of children with cryptogenic FIRES is less well understood.^6^ Here we observe elevated serum IL‐6 levels as reported in the literature^6^; and increased IL‐10 levels that have not been described previously. The apparent temporal alterations of the IL‐6 and IL‐10 levels potentially indicate a dynamic serum cytokine expression, which may reflect the clinical progression including responses to anakinra.

There are several limitations to this study. Although we were able to observe a temporal association between a decreased need for anesthetic infusions and anakinra initiation, we could not ascribe a causal effect to anakinra. Several cEEG studies were unavailable for the analyses which may affect the validity of the EEG findings. The timing and frequency of the serum CRP and cytokine studies were variable in relation to the seizure onset and anakinra initiation, which precludes more systematic analyses of the effects of refractory status epilepticus and anakinra on the systemic inflammatory changes. Because many systemic factors can influence inflammatory markers such as CRP and cytokines, the observed changes may not reflect the underlying neurological condition but an epiphenomenon of the overall severity of illness. The small sample size of our cohort precluded correlational analyses.

Despite the limitations, this study highlights a potentially active systemic inflammation in cryptogenic FIRES that merits further investigation. The candidate EEG and serum inflammatory biomarkers require future studies to assess their utility for case refinement and targeted immunomodulation for children suspected of FIRES/NORSE.

## Acknowledgments

We would like to thank the healthcare providers for their thoughtful care of the patients; and the families for their determination.

## Author Contributions

Dr. Yi‐Chen Lai conceptualized and designed the study, analyzed and interpreted the data, drafted, and revised the manuscript. Drs. Gabriella Abou‐El‐Kheir, Thao Nguyen, and Hanerhoff collected the data and revised the manuscript. Dr. James Riviello conceptualized the study, interpreted the EEG, and revised the manuscript. Dr. Eyal Muscal conceptualized and designed the study, and revised the manuscript.

## Conflict of Interest

Dr. Muscal served on the advisory board for Swedish Orphan Biovitrum (Sobi). Other authors do not have any conflict of interest to disclose.
